# Creating healthy remote store food environments: use of the Store Scout App in practice

**DOI:** 10.1186/s12889-025-23251-9

**Published:** 2025-07-03

**Authors:** Amanda Hill, Meaghan Christian, Gina Absalom, Brianna Sanderson, Hannah Downes, Sheida Navidi, Anna Murison, Emma McMahon, Grace Dwyer, Julie Brimblecombe

**Affiliations:** 1 Department of Nutrition, Dietetics & Food, Monash University, Notting Hill, VIC 3168 Australia; 2 Primary and Public Health Care, Central Australia, NT Health, Northern Territory, Alice Springs, 0870 Australia; 3 Outback Stores, Northern Territory, Berrimah, 0828 Australia; 4 Menzies School of Health Research, Charles Darwin University, Darwin, NT 0810 Australia; 5 Faculty of Health, Charles Darwin University, Northern Territory, Darwin, 0810 Australia

**Keywords:** Store Scout App, Store survey, Food retail, Food environment, Public health nutrition, Remote stores

## Abstract

**Background:**

The retail food environment influences food options and purchasing behaviour. Nutrition professionals can support food retailers to create health-enabling food environments. The Store Scout App (Store Scout) is a decision-support tool designed to facilitate this - it rapidly assesses health-enabling store practice and provides real-time feedback to retailers. This research aimed to assess the use of Store Scout by nutrition professionals in their practice to support retailers further their adoption of best-practice for community health improvement.

**Methods:**

Store owners of 30 stores in Aboriginal remote communities of Central Australia were invited to participate. Nutrition professionals servicing these stores received online training in creating healthy remote stores and access to Store Scout. Store appraisals using Store Scout were conducted from March 2021-August 2022. Practice change was assessed quantitatively using practice scores out of 100 generated by Store Scout for seven food categories and overall (baseline and follow-up). Usability was assessed qualitatively using semi-structured interviews with the nutrition professionals based on the Modified System Usability Scale. Thematic analysis was performed using Gale’s framework method for qualitative data analysis.

**Results:**

Fourteen of sixteen consented stores had appraisals completed at baseline, and 10 of the 14 stores at follow-up. Average overall practice scores increased from 60% (95%CI 53,67) at baseline to 64% (95%CI 56,73) at follow-up. Nutrition professionals (*n* = 4) found Store Scout quick and easy-to-use. The App helped facilitate effective collaboration with remote retailers. The ability to provide instant feedback and actionable evidence-based suggestions was an asset. Key recommendations to improve usability were identified.

**Conclusions:**

Store Scout is useful in practice to enhance engagement between nutrition professionals and retailers and shows potential to support adoption of healthy food retail best-practice and monitoring for continuous improvement. Its potential to support healthy food retailing in non-remote settings warrants investigation.

**Supplementary Information:**

The online version contains supplementary material available at 10.1186/s12889-025-23251-9.

## Background

### Retail food environments and health

The retail food environment, is well recognised as a setting that can influence food options and purchasing behaviour [1–3]. Health interventions in retail food environments can promote healthy food purchases and positively influence dietary outcomes [4, 5]. Globally however, retail food environments preference the marketing of unhealthy food and beverages, which drives unhealthy food purchasing [3, 6], thereby contributing to the rising prevalence of obesity and related preventable chronic disease [7, 8]. This trend is apparent in Australia [9], and due to the ongoing effects of colonisation, its impact on traditional food ways, and political, economic and social inequities, Aboriginal and Torres Strait Islander Australians, especially those living remotely, experience a disproportionate burden of preventable chronic disease in comparison to non-Indigenous Australians [10].

### Health-enabling remote community stores

There are over 230 food retail stores servicing Aboriginal and Torres Strait Islander communities in remote Australia [11]. Unique to this setting, approximately 40% of these stores are owned by Indigenous corporations [11], meaning they have a community-elected board of directors. Stores may be managed independently (54%) or by a store management company (46%) [11]. Aboriginal and Torres Strait Islander store directors have recognised the critical role of the community store for health improvement and led bold in-store initiatives to modify product availability and restrict placement and promotion of unhealthy food [12–16]. These initiatives have been shown to be effective in increasing healthy food purchases and reducing sales of unhealthy foods and added sugar purchased in food and drinks [12–16]. As reduced consumption of added sugars is associated with decreased body weight [17, 18], reduced risk of preventable chronic disease [18] and reduction in dental caries [19], supporting communities to create and sustain health-enabling retail food environment initiatives is likely to have significant positive flow-on effects for dietary intakes and overall community health.

In the remote context of Australia, nutrition professionals are well positioned to facilitate the co-design of health-enabling strategy with stores [20]. In the Northern Territory (NT) of Australia where this study took place, the nutrition workforce is employed by government and Aboriginal community-controlled organisations, and may involve a dual clinical dietetics and public health nutrition role that includes a nutrition service to remote stores [21]. Despite this key role, limited access to store-specific professional development, training resources and decision-support tools have been identified as key barriers for nutrition professionals [20].

### The Store Scout App

The Store Scout App (Store Scout), developed by Menzies School of Health Research, is a decision-support tool for use by nutrition professionals to rapidly assess in-store health-enabling practice and provide real-time feedback [22, 23]. Its development was in response to the need for an objective appraisal and feedback tool and mechanism to communicate evidence-based practice with retailers [23]. Store Scout assesses how both healthy and unhealthy products are marketed within-store using the ‘4Ps of marketing’ (Product, Placement, Price and Promotion). At the time of its development there was no other tool available globally that assessed all 4Ps of marketing and provided a rapid feedback function [23].

Validation found Store Scout to have very good inter-rater reliability, high agreement between scores and high internal consistency [22] and indicated its potential as a benchmarking and continuous improvement tool.

Store Scout has been used successfully in research settings and shown potential for use by nutrition professionals through pilot-testing [22, 23], however use and impact of Store Scout as part of their usual practice has not been formally tested.

### Study aims

We aimed to: (i) determine whether Store Scout (together with healthy food retail training), when used by nutrition professionals in their usual practice, facilitates a co-design approach with food retailers to adopt best-practice health-enabling strategies; and (ii) assess usability and acceptability of Store Scout as a healthy food retail continuous quality improvement tool for nutrition professionals.

This research seeks to inform the integration of Store Scout into remote nutrition practice to support store directors and store operators in their further adoption of best-practice in food retail for community health improvement.

## Methods

### Study design

This study was conceptualised in response to a request from nutrition professionals at NT Health, Primary and Public Health Care, Central Australia to use Store Scout as part of their usual practice. Mixed methods were used involving (i) quantitative data (using a before and after cross-sectional design) to assess adoption of best-practice, using Store Scout practice scores at two time points; and (ii) qualitative data to assess the usability and acceptability of Store Scout from a user’s perspective, collected through semi-structured interviews with nutrition professionals. The study is reported according to the requirements for Standards for Reporting Qualitative Research (SRQR) [24].

### Study context

The research was conducted in collaboration with NT Health, Primary and Public Health Care, Central Australia and Outback Stores Pty Ltd. Outback Stores Pty Ltd is Australia’s largest remote retail management organisation, a commonwealth government entity with an independent board that supports 58 remote stores across Australia, including 19 stores in the Central Australia and Barkly regions of the NT [11]. These stores operate in environments isolated from urban centres, with goods freighted from suppliers over long distances by road, rail, sea or air [25].

NT Health, Primary and Public Health Care, Central Australia employs nutrition professionals in dual clinical dietetics and public health nutrition roles. At the time of this study, there were seven permanent positions to service 27 remote communities and outstations (smaller homeland communities) across the Central Australia and Barkly regions, each with populations ranging from 50 to 1000 residents and located between 120 and 750 km (1–8 hours’ drive) from the nearest regional centre. These are large geographic regions covering over 800,000 km² in the NT of Australia [26, 27].

Nutrition professionals who work with these communities provide nutrition support to external services such as community stores. Support provided may include: store staff capacity building in health and nutrition; development, review and/or evaluation of a store’s nutrition policy; healthy product range and placement advice; and facilitating in-store education and promotional activities such as shelf labelling, taste testing or cooking demonstrations [20, 21]. The relationship between nutrition professionals and community food retailers is quite unique to the remote Australian context, facilitated by having a dedicated remote nutrition workforce in the NT for over two decades [21].

The Remote Community Stores Licencing Scheme under the Stronger Futures in the Northern Territory Act 2012 (sunset 2022) [28], at the time of this study, provided a regulatory background for nutrition professionals to engage with remote food retailers. This Scheme mandated minimum food security standards for remote community stores (including having a store nutrition policy, stocking an adequate range of healthy foods, promoting healthy foods and implementing healthy food practices within-store). While remote nutrition professionals did not have authority to enforce Scheme standards, they could be referred to if Scheme enforcement officers identified a store required nutritionist support.

In remote Australia, community-owned stores have a community-elected board of directors, in contrast to privately-owned stores that may not have community input into their operations. The board of directors or private owner may either contract an independent manager (independently-managed store) or a company (such as Outback Stores Pty Ltd) to manage store operations (store management company managed store). This study incorporates stores from these various store ownership and management structures.

### Sampling and recruitment

#### Remote stores

Nutrition professionals from NT Health, Primary and Public Health Care, Central Australia, invited the Aboriginal and Torres Strait Islander store directors and other store owners of all 30 community stores servicing the 27 communities and outstations in Central Australia and the Barkly regions to participate. Recruitment was completed face-to-face where possible or via video presentation emailed to the store manager. Store owners were asked for their consent to (i) allow a nutrition professional to complete Store Scout in their store at two timepoints, (ii) receive feedback and a score for their store based on promotion, price, product and placement practice, and (iii) co-design an action plan with the nutrition professional to improve practice. Consent procedures used comply with the National Health and Medical Research Council Ethical Guidelines for Research with Aboriginal and Torres Strait Islander Peoples [29].

#### Nutrition professionals

Four of the six nutrition professionals employed at NT Health, Primary and Public Health Care, Central Australia at the time of the study serviced the consented stores and were invited to participate in a semi-structured interview about their experience with use of Store Scout.

#### Ethics

Ethics approval was granted from the Central Australian Human Research Ethics Committee Centre for Remote Health (CA-20-3750) and the Monash Human Research Ethics Committee (30761). Principles of Reciprocity, Respect, Equity, Responsibility, Cultural Continuity, Spirit and Integrity in relation to Aboriginal and Torres Strait Islander Research as set out in the NHMRC Ethical Guidelines [20] were addressed in the ethics application.

#### Intervention

Nutrition professionals at NT Health, Primary and Public Health Care, Central Australia were provided with training in creating healthy remote store food environments through an interactive online course and in use of Store Scout.

*“Creating a Healthier Remote Store Food Environment” online course*.

The online course was designed by Monash University with Health and Wellbeing Queensland to upskill participants in their ability to support health-enabling practice in remote stores, in line with the Healthy Stores 2020 Policy Actions [21]. The course covered eight key topics (see Table 1). Each topic included a short expert presentation and a case-study group discussion.

**Table 1:** Key topics covered “creating a healthier remote store food environment” online course

Session topics	Brief Description
1. Food retail partnerships	Establishing a relationship with food retailers through best practice communication and engagement approaches
2. History and business models	The historical role of remote stores in health improvement and the different business models of this setting
3. Takeaway	Supporting takeaways to promote nutrition and sell healthy food
4. Fruit and vegetables	Strategies to promote the sale of fruit and vegetables
5. Discretionary Items (Focus on Sugary Drinks)	Strategies to reduce the promotion of discretionary items in the retail setting and achieve decreased sales of these items
6. Store Networks	The store social network and identification of the key external entities who influence store operations and decision making
7. Co-design	Working with the food retail sector– Co-design of best practice strategies and nutrition policy
8. The Store Scout App	Using Store Scout App as a co-design and benchmarking tool

### Store Scout App (version 1)

Store Scout is a validated tool to promote evidence-informed practice which aims to restrict visibility and reduce sales of discretionary products and to increase visibility and boost sales of targeted core foods [14, 15]. It assesses the use of the 4Ps marketing principles for different foods and beverages in-store, including: Product (availability and range), Placement (visibility, accessibility, proximity to high-traffic areas), Price (discounts, deals and/or subsidies), and Promotion (such as labelling, signage, displays or demonstrations) [15].

On completing a full assessment in-store, Store Scout gives an overall practice score and a score for each of seven different food categories (Fruit & Vegetables, Drinks, Snack Foods, Meals & Convenience Foods, Breads & Cereals, Meat & Seafood and Dairy & Eggs) [22]. Scores range from 0 to 100, with higher scores indicating a more health-promoting store. These scores are calculated for each category as a percentage of the total possible points and then averaged across the seven categories to provide an overall percentage score. A detailed description of Store Scout scoring has been published elsewhere [22]. Store Scout provides a summary of suggested best-practice strategies (e.g., removing confectionery from the front counter, or making water visible at the front of the store) to facilitate discussion with the business owners and managers on what changes might be feasible and acceptable for their store to implement to become more health-enabling [22].

A store manager survey asks how the 4Ps of marketing are considered in the store’s operations and their view on the importance of different nutrition-related practices (e.g., having a store nutrition policy, linking with other local services to promote health, having a store environment that influences healthy options).

### Use of Store Scout App to support continuous quality improvement in remote stores

Store Scout was designed with built-in action planning features for use in quality improvement. The following co-design process was determined by the researchers with the nutrition professionals: Following the in-store appraisal, research team provides the nutrition professionals with a report of the Store Scout scores, three strongest areas of health-enabling practice and suggested actions for practice improvement. Nutrition professionals discuss the results and report with store owners and/or store operator and co-design an action plan with one to three evidence-based actions to implement.

### Data collection and analysis

#### Adoption of best-practice

Nutrition professionals used Store Scout to assess in-store practice at two timepoints, approximately six months apart during 2021 and 2022 (baseline and follow-up). Descriptive statistics (mean, standard deviation and 95% confidence interval) were used to describe aggregated Store Scout practice scores and mean comparison tests (paired t-tests) were performed to explore change in scores between baseline and follow up with *p* < 0.05 used as the cut-off for statistical significance. All statistical analyses were conducted in Stata (version 17 SE). Stores that completed Store Scout Appraisals at baseline and follow-up were included in the main analysis. Differences between completers and non-completers were explored using a one-way ANOVA.

#### Store Scout App usability and acceptability

Usability and acceptability of Store Scout was assessed qualitatively, using a semi-structured interview based on the Modified System Usability Scale [30]. The interview comprised three sections: (i) open-ended questions on App user experience, its functions, and ease-of-use; (ii) questions on what participants liked least and best about the App; and, (iii) description of a user experience with a store operator and/or owner. The interview guide is provided in the Supplementary Table 1.

Four participating nutrition professionals were interviewed by MC in May 2022 after they had used Store Scout at baseline. Interviews were conducted via Zoom technology. All interviews were recorded and secured electronically. The interviews were transcribed verbatim by an independent transcription service.

The authors applied a constructionist perspective allowing for the experiences and world views of both the researchers and participants (nutrition professionals) in the construction of new knowledge.

The interviews were analysed by AH and MC using Gale’s framework method for analysis of qualitative data [31]. This involved deductive and inductive coding, and iterative development of a thematic framework using elements of the Modified System Usability Scale as well as inductively derived codes. AH and MC both open coded 25% of the transcripts, and preliminary codes were discussed and debated. Remaining transcripts were then open coded by AH using NVivo (version 14). AH familiarised themselves with the data by reading and re-reading the interview transcripts and listening back to original audio recordings. The analytical (coding) framework was finalised with MC then applied by AH to all transcripts in NVivo. The data were then summarised in a framework matrix in Microsoft Excel (Version 1808), and themes generated corresponding to (i) use of Store Scout in practice and (ii) usability and acceptability.

#### Researcher reflexivity

The authors include four researchers, the four nutrition professionals employed by NT Health, Primary and Public Health Care, Central Australia who used Store Scout and two store group nutritionists with Outback Stores, who combined have extensive experience working in remote settings. One author is Indigenous, and all have an interest in supporting improved nutrition outcomes in Aboriginal and Torres Strait Islander communities through equity and capacity strengthening approaches. We believe that equitable access to good quality, nutritious and culturally appropriate food is a human right and that a contribution we can make as non-Indigenous researchers to address health inequities is to strengthen the access to evidence for Aboriginal and Torres Strait Islander leaders to inform the decisions they need to make for the health of their communities.

At the time of the study, 70% of the authors were based in the NT and worked with remote community stores in an outreach capacity. Despite the collaborative approach, there may still be a power imbalance between researchers and nutrition professionals which could have led to positive bias in qualitative interviews. A strength of including these motivated and engaged nutrition professionals as co-authors is the in-depth insider knowledge of service delivery in the unique remote Australian context and how this can impact the use of Store Scout.

## Results

Of the 30 invited stores, 17 stores (57%) across 13 remote Aboriginal communities and outstations in the Central Australia and Barkly regions agreed to participate. Non-consenting stores either lacked capacity to participate, had recently changed management or a response was not able to be attained in the allocated timeframe for recruitment.

Following recruitment, two of the participating stores merged into one business, resulting in a total of 16 participating stores. Store Scout baseline appraisals were conducted in 14 of the 16 stores between March-April 2022, as two stores were unable to be appraised due to no nutrition professional available to collect data for that region (position vacant), and limited store trading hours. Follow up appraisals were completed in 10 of the 14 stores between August-September 2022, as two stores withdrew from the study (due to selling the business and reduced capacity), and two stores had no nutrition professional available to collect data for that region (position vacant). Table 2 presents the store ownership and management characteristics together with appraisal completion at each timepoint for each of the 16 participating stores. Appraisals occurred between 4 and 5 months apart (average 4.5 months).

**Table 2:** Store ownership and management characteristics with appraisal completion

Store Characteristics	Baseline, *n* (%)	Follow up, *n* (%)	No data collected, *n* (%)
Community owned, managed by store management company	4 (29%)	4 (40%)	0
Community owned, independently managed	3 (21%)	0	0
Privately owned, independently managed	7 (50%)	6 (60%)	2 (100%)
TOTAL	**14**	**10**	**2**

### Adoption of best-practice

There was an increase in the aggregated overall Store Scout score and in practice scores for all food categories from baseline to follow-up as shown in Table 3. Supplementary Table 3 provides overall and food category scores for each store from baseline and follow-up.

The overall average practice score increased from 60% (95%CI 53,67) at baseline to 64% (95%CI 56,73) at follow up. Snack foods showed the largest practice change, increasing from an average of 42% (95%CI 33,50) for all stores combined to 49% (95%CI 40,59). The least change was shown for the fruit and vegetable category. Differences in baseline to follow-up scores did not reach statistical significance.

For each Store Scout food category, except dairy and eggs, the four non-completer stores had lower mean baseline scores compared to the 10 completer stores. This difference reached statistical significance for overall practice, fruit and vegetables, drinks, and meals and convenience category scores (Supplementary Table 2).

**Table 3:** Change in mean store scout practice scores, at baseline and follow-up

	Baseline (*n* = 10)			Follow-up (*n* = 10)			Baseline to Follow-up change			
	Mean (%)	SD	95%CI	Mean (%)	SD	95%CI	Mean Diff	SD	95%CI	*P* value
**Overall Practice Score**	**60**	9.7	53, 67	**64**	11.8	56, 73	**4.6**	9.0	-2, 11	0.14
Fruit & Vegetables	**71**	12.3	63, 80	**73**	18.9	60, 86	**1.6**	8.6	-5, 8	0.57
Drinks	**66**	17.4	53, 78	**70**	17.2	58, 83	**4.9**	14.0	-5, 15	0.29
Snack Foods	**42**	11.9	33, 50	**49**	13.3	40, 59	**7.4**	14.7	-3, 18	0.14
Meals & Convenience Foods	**45**	12.2	36, 54	**49**	17.3	37, 62	**4.3**	18.2	-9, 17	0.47
Breads & Cereals	**66**	15.1	55, 77	**72**	18.7	59, 85	**6.0**	15.2	-5, 17	0.24
Meat & Seafood	**68**	12.0	59, 76	**70**	9.0	63, 76	**2.1**	14.3	-8, 12	0.65
Dairy & Eggs	**61**	21.8	46, 77	**67**	14.9	56, 78	**5.6**	18.5	-8, 19	0.36

### Use of Store Scout App in remote nutrition practice to facilitate health-enabling food retail

Five themes were generated in relation to participants’ views on use of Store Scout in their usual practice.

### Enhancing collaboration with remote retailers

Multiple benefits of using Store Scout to enhance how nutrition professionals engage with remote stores were reported, including building of relationships and fostering collaboration.

The App was thought to be helpful as a *“first point of call*,* building that strong relationship between the store and the nutritionists”* (Participant 1). Participants felt the App supported them in *“opening up discussions and leading in what you can continue doing”* (Participant 1) with store operators, as well as providing a structure for framing these discussions: *“I find like having that conversation with her easier– using that framework– that structure*,* because then we just could focus on what we could do”* (Participant 2).

### Engagement

Participants noted the buy-in required at the store level from store management for Store Scout to be used successfully: *“I think if you haven’t got a store manager who’s interested in doing it*,* it’d be a real challenge. So as much as I think it’s a very good tool*,* you really do need the buy-in from the store*,* make any changes and build that relationship.”* (Participant 1).

They also recognised benefits for sharing their use of Store Scout with Store Directors and the wider community:

*“the more people know about this project and how it will benefit the store*,* the better*,* especially if they are living in the community and it’s their store– that they can advocate for [a healthy store]”* (Participant 2).

#### Evidence-base, objectivity and practicality

Store Scout was thought to be a practical tool for working with remote stores, and provided objectivity, a best-practice evidence base and ready access to practical strategies for a healthy store. Instant feedback on results was seen as an asset of Store Scout: *“I really like that it’s got the action plan straight at the end and the scores right at the end…having that ability to have that discussion straight off the bat is really helpful.”* (Participant 1).

*“the little report bit that it spits out at the end with the percentages is also really easy to chat through with store managers. Or it will start a discussion*,* at least.”* (Participant 4).

*“I find that that section of the strategies of the Store Scout very helpful… I ran through the list with [the store manager]… And then she went through it and had a look*,* and she picked up a few things that she wanted to change”* (Participant 2).

*“as a dietitian who has relationships on the ground*,* it’s really good to be able to offer something more objective rather than coming in and*,* I suppose*,* having a more subjective approach to what the store should be doing.”* (Participant 4).

### Supporting co-design of action plans

Store Scout food category practice scores helped users prioritise key action areas with store operators: *“We just had a chat about the… categories that they scored the lowest in and some example strategies they could implement from that.”* (Participant 3), and allowed for positive reinforcement of areas of strength, as *“people were interested to see the scores and how their store did well”* (Participant 4).

The wide range of suggestions for action that the App provided was a positive: *“I like the range of strategies that it provides as well. So there are things such as putting up a poster*,* which is really easy for a store to do*,* to the strategies that are harder to implement. So I think it’s good to have that scale*,* particularly for some of the stores that are more difficult to engage in nutrition work… it’s just a starting point with them.”* (Participant 4).

*“So she [store manager] had a kind of planogram that she couldn’t change*,* so we just didn’t touch that. But… there were other things that she [store manager] was - she [store manager] almost was excited to change some of the things.”* (Participant 1).

### Monitoring and evaluation

Nutrition professionals saw potential for small initial changes to build over time with repeated use of the App: *“I think it’s good… that the plan is for it to be done and then done again because you can build on those strategies”* (Participant 1).

*“something that we can evaluate*,* and like keep going back and see how the stores have improved.”* (Participant 2).

### Store Scout App usability and acceptability

Six key themes were identified on usability and acceptability of Store Scout.

### Ease of use

Store Scout was described as *“intuitive”*,* “self-explanatory”*,* “simple”*,* “straight-forward”* and *“user-friendly”.* The logical structure of the App and access to tooltips contributed to this.

Participants felt Store Scout could be used by others including health promotion officers, store managers, Aboriginal health workers and community members: *“it is really helpful having a nutritionist involved*,* where practically possible… I don’t see why non-nutritionists and non-health professionals couldn’t use the App”* (Participant 1).

*“most people would be capable of using the App… in the stores*,* showing local workers how to use it would be really*,* really useful.”* (Participant 3).

### Time to complete

Store Scout was described as *“really quick to use”* (Participant 4) and the time taken to complete the appraisal was considered appropriate. This was dependent on user familiarity with the App and store layout, with smaller and more familiar stores being quicker to complete. Participants were conscious of time burden on store operators, and saw it as positive that Store Scout *“didn’t take up too much of the store managers’ time”* (Participant 4).

### Functional enhancements

Participants noted specific Store Scout functions that could be enhanced, including saving, editing and then submitting a completed appraisal.…to *“see that these are the [things] previously done with that store… would be really helpful.”* (Participant 1) It was suggested that to *“…have an option to sort of save it and know that it’s within a draft form”* (Participant 1) would be useful particularly with interruptions when doing the appraisal: *“It’s just something that you need to have*,* because our work is just*,* sort of*,* changing all the time– Like*,* you need to step out of the store*,* or go in. And so*,* having the option to save data for each part that they have done*,* and being able to go back to it*,* without losing it– That’s why it’s very important.”* (Participant 2).

### Store Scout App question enhancements

Participants identified the more subjective Store Scout questions as difficult to answer (such as whether certain products are ‘well stocked’, or have a ‘limited range’ or ‘limited shelf space’): *“If you didn’t work in a remote community*,* it’s kind of up to interpretation if something’s well stocked*,* if they’ve got a variety*,* because it’s very*,* very different where you are.”* (Participant 3).

*“It sort of becomes more subjective in what the nutritionists think at the time*,* and then impacts on the accuracy of the data that’s collected.”* (Participant 2).

Lack of familiarity with retail terms was thought to possibly cause confusion if not defined adequately: *“I know that they are asking for different things*,* like the shelf space is the facings and the range is how many different items they have. But I suppose if you’re not that familiar with the terminology*,* they kind of do sound like they’re asking the same question.”* (Participant 4).

Adding an option ‘not applicable’ for questions not relevant to that store was suggested: *“one thing I didn’t like was having questions that weren’t applicable to the store that I was in and not being able to answer that… I felt like that was disadvantaging stores by the fact that they just - they don’t have the product*,* but you still have to answer no.”* (Participant 3).

### Action plan enhancements

Participants identified enhancements for the action plan feature to prioritise actions and streamline feedback: “*some of the stores we were working with had pretty low scores*,* so the amount of suggestions in the action plan are quite long”* (Participant 1)

*“it would be good to maybe have categories [in the action plan] to be able to streamline your discussions a bit more.”* (Participant 4).

*“a way to identify some of the action plans which are going to… potentially give more bang for buck… So possibly showing within the App so you can have that discussion with the store manager what’s going to make a bigger difference.”* (Participant 1).

### Store manager survey enhancements

Participants found some of the store manager survey questions *“a bit clunky”* (Participant 1), *“a bit confusing and subjective”* (participant 2) or *“a bit tricky to ask”* (Participant 4). Suggestions included providing a preamble, clear definitions of terms used and improved question flow.

## Discussion

This research addresses an evidence gap on the effectiveness of interventions that aim to change the practices of practitioners and retailers who support and shape retail food environments. Store Scout was designed to be used in research and practice [22] and this mixed methods research suggests that the Store Scout App is acceptable to implement as a continuous quality improvement tool as part of nutrition practice in a remote setting. Results suggest that using Store Scout (with training in co-design of healthy remote store food environments) can enhance engagement and help build relationships between nutrition professionals and retailers and provide structure to co-design action plans and facilitate adoption of best practice in remote food retail.

Previous research has identified a need for a valid tool to measure change within the store food retail environment, which reflects the different types of food settings [32, 33]. The co-design process associated with use of Store Scout brings together evidence-based healthy retail suggestions for action based on the 4Ps of marketing, and the expertise of store operators and/or store directors and nutrition professionals to determine feasible, financially viable and acceptable ways to improve nutrition-related practices within the store. Evidence-based practices, such as the promotion and prominent placement of healthy foods, play a pivotal role in supporting customers in making healthy food purchases. Furthermore, research has shown that this co-creative process in use of Store Scout App that facilitates a mutually-acceptable pathway for store adoption of best-practice, is more likely to have success from both a business and health perspective [34, 35].

Previous research has identified limited access to specific training and decision-support tools as barriers for nutrition professionals in engaging effectively with stores [20]. Our findings confirm that Store Scout can fill this gap as a key tool for supporting monitoring and continuous quality improvement. Participants felt that while nutrition expertise was useful in using Store Scout App, with appropriate training store operators and or staff would be able to use Store Scout. Self-assessment can play a role and provide timely feedback to store operators, however independent assessment at least once a year would provide an objective and comparable measure. The benefits of training users living in remote communities, such as store staff and/or Aboriginal health workers, was highlighted as beneficial for building local capacity and advocacy for nutrition, especially as not all communities have access to a visiting nutrition professional.

Store Scout user experience was consistent with pilot testing of the Healthy Store Environment Tool (precursor to the Store Scout App) [23], where time taken to complete Store Scout was acceptable, Store Scout assisted with feedback and facilitated discussions on best-practice and was relevant to the nutrition professionals practice. The instantaneous results the Store Scout App provides after assessment was valued, as it enabled immediate feedback and identified actionable best-practice suggestions.

In line with previous findings [16, 23, 36], participants noted that the ‘buy-in’ from store operators was critical for the effective use of the App and allowed a starting point and opportunity for discussion for stores with low engagement.

Our findings, though based on a small sample and limited to two time-points, align with McMahon et al. [22], demonstrating Store Scout’s effectiveness in capturing changes in store practices over time, supporting its use as a monitoring tool. Outside of research projects, the Store Scout App has not previously been used to monitor change in store environments, and few evidence-based tools have been used for this purpose in remote and non-remote food retail settings. This was the first time these stores engaged with Store Scout and its associated co-design process. Despite modest and statistically non-significant changes in practice scores, the positive changes observed across food categories suggest an increase in adoption of health-enabling retail practice. Store Scout would likely have greater impact over time as users and retailers and communities build trust and confidence to adopt further best-practices. In healthcare, repeated audits and feedback from trusted sources with clear goals have been shown to encourage evidence-based practices [37]. Similarly, a study involving four remote Aboriginal communities in Australia, found that providing feedback on practice performance to store directors and managers, along with action planning, led to positive change in store practice [14, 38].

There is potential for the Store Scout App to assist with store nutrition policy execution, as actions included in Store Scout are best practices which could be expected in store nutrition policies in remote communities in Australia. It is through monitoring and implementation of organisational policies and procedures that continuous improvement for health-enabling food retail can be facilitated for the communities they serve.

### Strengths and limitations

This study is based on one specific health service and region of the Northern Territory (although vast in size), hence the small number of interviewees. Our results from testing Store Scout in a remote practice setting suggest it could be usable and acceptable within the workloads of remote nutrition professionals in other areas of Australia, in line with the original intent of its design [23]. A strength of this research is the diverse range of stores that participated in the project, including community-owned stores managed by a store management company, community-owned and independently managed stores, and privately owned and independently managed stores. This suggests that using the Store Scout App as a continuous quality improvement tool is acceptable to partners working with different store management and governance structures in remote communities in the NT.

The COVID-19 pandemic impacted this study, causing delays in data collection during 2021 due to travel restrictions, which led to a shorter gap between Store Scout evaluation timepoints.

Our mixed methods approach quantifies changes in the food retail environment with the Store Scout intervention while capturing user perspectives to inform its large-scale uptake. Managed by Monash University, the Store Scout App can continue to be updated to align with current food retail policies and initiatives. This study applied a co-design process utilising the action plan component in the Store Scout App, meaning for community-owned stores, the decision-making is in the hands of the community. The findings from this project demonstrate that Store Scout can aid the nutrition workforce to co-design healthy food retail strategies with the community to ensure evidence-informed strategies are feasible and acceptable.

### Future implications

The findings from this research provide evidence that use of Store Scout in remote nutrition practice can support the co-design of health-enabling food retail initiatives between nutritional professionals and remote retailers across various store governance structures. The Store Scout App offers a structure to assess the food retail environment and fosters collaborative relationships. Designed to reliably assess current practices, the co-design process used evidence-based suggestions to improve the healthiness of the retail food environment. This research also highlights the importance of a respectful, trusting and collaborative process between nutritional professionals, store owners, managers and community members in remote contexts.

While Store Scout was initially designed for remote Australian Aboriginal and Torres Strait Islander community stores, it has also been tested in metropolitan food retail stores [22]. Its potential to assess, monitor and promote continuous quality improvement in urban and rural settings warrants future research. Independent retailers may be more likely than large supermarket chains to engage with Store Scout, as smaller retailers often show greater concern for their community [35, 39]. In remote Australia, community-led governance and supportive store nutrition policies facilitate decision-making [16]. Outside this context, retailers’ autonomy in merchandising and negotiating with suppliers may differ [40]. However, there is evidence that retailers do not identify suppliers as barriers to implementing healthy retail initiatives [41], and retailers retain decision-making power despite supplier influences [42].

The results provide valuable insights into improving the Store Scout App's functionality and usability. Nutrition professionals suggested enhancing data storage, such as incorporating the ability to see all completed appraisals for each store to explore changes over time. Additionally, providing a clear structure and categorisation within the Action Plan section would allow targeting suggestions by food category scores, making appropriate actions easier to select, especially where stores achieve lower scores and have many suggestions to review.

The tooltips (supportive text) in the Store Scout App could also be improved. Participants expressed difficulty answering questions requiring subjective judgements, such as whether products were ‘well stocked’, had a ‘limited range’ or ‘limited shelf space’. Although this only encompasses only 10 of the 199 questions, clearer definitions of terms like ‘shelf space’, ‘limited range’ or ‘well stocked’ in the tooltips, and more objective guidance, could enable more repeatable responses among users.

Since the development of Store Scout, the International Network for Food and Obesity/non-communicable disease Research, Monitoring and Action Support (INFORMAS) has developed indicators to monitor food environments, policies, and actions. The Food Retail Module– Food Availability in Supermarkets Protocol details an internationally standardised measurement methodology of the supermarket environment, including availability, prominence and promotion of healthy and unhealthy products [43]. This protocol requires equipment to measure linear shelf length allocated to products and calculation of shelf space and healthy to unhealthy product ratios [43]. The Nutrition Environment Measures Survey in Stores (NEMS-S), assesses the nutrition environment in food stores, evaluating the availability, pricing, and quality of healthier versus less-healthy food options [39]. In contrast, Store Scout evaluates how health-enabling the food store environment is against evidence-based best practice across all 4Ps of marketing (product, placement, price and promotion) in a shorter timeframe [22, 44]. It is comprehensive, but does not require equipment or user calculations like the INFORMAS Food Retail Module. Despite some subjective judgements, most questions in the Store Scout App (79%) have good to very good inter-rater reliability [22]. The Store Scout App was designed to be easy-to-use with basic training and provide instant feedback to motivate best-practice uptake.

Future updates of Store Scout will include these improvements to enhance the user experience.

## Conclusions

Store Scout can enhance engagement between remote nutrition professionals and retailers and has potential to increase evidence-based health practice in stores in remote communities of Australia. This approach acknowledges the expertise and knowledge of Aboriginal and Torres Strait Islander store-owners to determine feasible and acceptable ways to enable healthier food purchases in remote stores. The potential for Store Scout to be used in other settings warrants investigation.

## Electronic supplementary material

Below is the link to the electronic supplementary material.

Supplementary Material 1

## Data Availability

No datasets were generated or analysed during the current study.

## Abbreviations

Store Scout App: Store Scout or the App
4Ps of marketing: Product, Placement, Promotion, Price
