# Supplementary material for: Creating healthy remote store food environments: use of the Store Scout App in practice
Source: BMC Public Health. 2025 Jul 3;25:2326. doi: 10.1186/s12889-025-23251-9 (PMC12224354; doi:10.1186/s12889-025-23251-9)
Supplement: Supplementary file 1 — Supplementary Material 1 [file 12889_2025_23251_MOESM1_ESM.docx]

**Supplementary Table 1: Modified System Usability Scale**

| Please describe your experience in your use of the App  Please describe the aspects of the App that were hard to use  Please describe the aspects of the App that were easy to use  How did you use the summary screens at the end of the App?  Do you think the App would be easy for other public health nutritionists to use?  Who else would you like to see trained to use this App and why?  Did you think the time taken to complete the App appropriate and why?  Can you describe how confident you felt in using the App?  What further information would you like to learn before using this App again?  What problems (if any) did you have in using the App?  What are your views about the look of the App?  What did you like least about the App?  What did you like best about the App?  Do you have any other comments about the App?  **Questions on use of App with store manager and store owners**  Please describe in detail your use of the App with the store manager and/or store owners?  Probe for – receptiveness of store manager/store owners, information shared, responses to information shared (positive and negative), actions you may have suggested and response to these, actions suggested by the store managers/store owners and your response to these, any follow-up action agreed on (i.e., discussion on what next)  How was this useful to you and your work?  Please describe in detail your use of the App data with others in the community and/or other stakeholders?  Probe for – who, for what purpose shared with others, how (in what way), responses (positive/negative), follow up action  How was this useful for you and your work? |
| --- |

**Supplementary Table 2. Difference in Store Scout Practice at baseline and follow up for completer stores**

|  | **Completed baseline data collection only (n = 4)** | | **Completed both time points (n = 10)** | | **P value** |
| --- | --- | --- | --- | --- | --- |
|  | **Mean (%)** | **SD** | **Mean (%)** | **SD** |  |
| **Overall Practice Score*** | **47** | 6.7 | **60** | 9.7 | 0.04 |
| Fruit & Vegetables* | **51** | 10.8 | **71** | 12.3 | 0.01 |
| Drinks* | **42** | 13.7 | **66** | 17.4 | 0.04 |
| Snack Foods | **39** | 1 | **42** | 12.0 | 0.59 |
| Meals & Convenience Foods* | **28** | 7.7 | **45** | 12.2 | 0.03 |
| Breads & Cereals | **51** | 13.1 | **66** | 15.1 | 0.11 |
| Meat & Seafood | **57** | 5.6 | **68** | 12.0 | 0.12 |
| Dairy & Eggs | **62** | 13.5 | **61** | 21.8 | 0.96 |

One-way-ANOVA, *p < 0.05

**Supplementary Table 3: Store Scout results for each store (baseline and follow-up)**

|  | Baseline | | | | | | | | Follow-up | | | | | | | |
| --- | --- | --- | --- | --- | --- | --- | --- | --- | --- | --- | --- | --- | --- | --- | --- | --- |
| Store | **Overall score** | **Fruit & Veg** | **Drinks** | **Snack Foods** | **Meals & Con** | **Breads & Cereals** | **Meat & Seafood** | **Dairy & Eggs** | **Overall Score** | **Fruit & Veg** | **Drinks** | **Snack Foods** | **Meals & Con** | **Breads & Cereals** | **Meat & Seafood** | **Dairy & Eggs** |
| 1 | 69 | 73 | 61 | 46 | 54 | 80 | 86 | 86 | 61 | 80 | 71 | 56 | 37 | 50 | 62 | 72 |
| 2 | 61 | 60 | 57 | 41 | 47 | 90 | 71 | 62 | 72 | 67 | 75 | 61 | 58 | 90 | 81 | 72 |
| 3 | 63 | 87 | 82 | 24 | 40 | 75 | 71 | 62 | 77 | 93 | 86 | 56 | 50 | 95 | 86 | 72 |
| 4 | 51 | 80 | 36 | 41 | 26 | 60 | 48 | 69 | 62 | 77 | 64 | 51 | 33 | 70 | 62 | 79 |
| 5 | 62 | 80 | 89 | 54 | 44 | 65 | 52 | 48 | 76 | 83 | 82 | 59 | 79 | 80 | 71 | 79 |
| 6 | 39 | 47 | 29 | 37 | 22 | 40 | 52 | 48 | - | - | - | - | - | - | - | - |
| 7 | 52 | 57 | 54 | 32 | 37 | 60 | 67 | 59 | 43 | 57 | 32 | 24 | 20 | 65 | 57 | 48 |
| 8 | 40 | 50 | 54 | 29 | 50 | 35 | 57 | 7 | 48 | 33 | 68 | 29 | 63 | 35 | 67 | 41 |
| 9 | 60 | 67 | 57 | 37 | 48 | 70 | 71 | 69 | 60 | 60 | 54 | 61 | 37 | 70 | 67 | 69 |
| 10 | 66 | 80 | 86 | 61 | 33 | 55 | 71 | 76 | 75 | 90 | 86 | 54 | 58 | 75 | 76 | 86 |
| 11 | 55 | 67 | 43 | 39 | 33 | 65 | 62 | 79 | - | - | - | - | - | - | - | - |
| 12 | 73 | 80 | 79 | 54 | 70 | 70 | 81 | 76 | 69 | 90 | 86 | 42 | 57 | 90 | 67 | 52 |
| 13 | 45 | 47 | 36 | 39 | 37 | 40 | 52 | 66 | - | - | - | - | - | - | - | - |
| 14 | 49 | 43 | 61 | 39 | 22 | 60 | 62 | 55 | - | - | - | - | - | - | - | - |
